# Minimally Invasive Mitral Valve Surgery Compared to Sternotomy in Patients Over 70 Years Old: A Retrospective Nationwide Multicentre Study in The Netherlands

**DOI:** 10.1093/icvts/ivag026

**Published:** 2026-01-28

**Authors:** Andrew Tjon Joek Tjien, Kinsing Ko, Samuel Heuts, Saskia Houterman, Maaike Roefs, Sjoerd Bouwmeester, Pim Tonino, Sandeep Singh, Robert Storm van Leeuwen, Jos Maessen, Peyman Sardari Nia, Niels Verberkmoes, Jules Olsthoorn, the Cardiothoracic Surgery Registration Committee of the Netherlands Heart Registration, S Bramer Amphia, R A F de Lind van Wijngaarden, B M J A Koene, J A Bekkers, G J F Hoohenkerk, A L P Markou, A de Weger, P Segers, D Stecher, R G H Speekenbrink, W Stooker, W W L Li, E J Daeter, N P van der Kaaij, Y L Douglas

**Affiliations:** Department of Cardiothoracic Surgery, Catharina Hospital, Eindhoven, 5623 EJ, The Netherlands; Department of Cardiothoracic Surgery, Radboud University Medical Center, Nijmegen, 6525 GA, The Netherlands; Department of Cardiothoracic Surgery, Isala, Zwolle, 8025 AB, The Netherlands; Department of Cardiothoracic Surgery, Maastricht University Medical Center, Maastricht, 6229 HX, The Netherlands; Cardiovascular Research Institute, Maastricht, 6229 ER, The Netherlands; Netherlands Heart Registration, Utrecht, 3511 EP, The Netherlands; Netherlands Heart Registration, Utrecht, 3511 EP, The Netherlands; Department of Cardiology, Catharina Hospital, Eindhoven, 5623 EJ, The Netherlands; Department of Cardiology, Catharina Hospital, Eindhoven, 5623 EJ, The Netherlands; Department of Cardiothoracic Surgery, Isala, Zwolle, 8025 AB, The Netherlands; Department of Cardiothoracic Surgery, Isala, Zwolle, 8025 AB, The Netherlands; Department of Cardiothoracic Surgery, Maastricht University Medical Center, Maastricht, 6229 HX, The Netherlands; Cardiovascular Research Institute, Maastricht, 6229 ER, The Netherlands; Department of Cardiothoracic Surgery, Maastricht University Medical Center, Maastricht, 6229 HX, The Netherlands; Cardiovascular Research Institute, Maastricht, 6229 ER, The Netherlands; Department of Cardiothoracic Surgery, Catharina Hospital, Eindhoven, 5623 EJ, The Netherlands; Department of Cardiothoracic Surgery, Catharina Hospital, Eindhoven, 5623 EJ, The Netherlands; Department of Cardiothoracic Surgery, Isala, Zwolle, 8025 AB, The Netherlands

**Keywords:** mitral valve surgery, minimally invasive mitral valve surgery, patients over 70 years old, nationwide registry

## Abstract

**Objectives:**

Older patients are more prone to postoperative morbidity and mortality after mitral valve (MV) surgery. Minimally invasive MV surgery (MIMVS) is increasingly adopted worldwide, with a potential benefit in the elderly. This study compares short-term and mid-term outcomes in patients above 70 years, undergoing MIMVS versus median sternotomy (MST), in a nationwide registry.

**Methods:**

All patients above 70 years undergoing primary elective MV surgery (±tricuspid valve [TV] surgery, atrial septal defect closure, rhythm surgery) between 2013 and 2021 were included. All data were extracted from the Netherlands Heart Registration. Primary outcomes were short-term morbidity, mortality, and 5-year survival.

**Results:**

In total, 1418 patients were included (MST *n* = 797, MIMVS *n* = 621). No statistically significant differences in baseline characteristics were found. Median Logistic EuroSCORE I was 6.3 [4.7–8.5] vs 6.0 [4.6–8.5], *P* = .27 for MST and MIMVS, respectively. Mitral valve repair (77.7% vs 64.7% *P* < .001) and concomitant TV surgery (43.9% vs 18.2%, *P* < .001) was more frequently performed in MST. Lower 30-day mortality was observed in MIMVS (0.6% [*n* = 4] vs 2.5% [*n* = 21], *P* = .01). Furthermore, the incidence of pneumonia, prolonged intubation, readmission to intensive care unit, kidney failure, and new-onset arrhythmia were lower for MIMVS. No difference in 5-year survival was found (MST: 89.1 ± 4.6% vs MIMVS: 91.6 ± 4.7% Log-Rank *P* = .51).

**Conclusions:**

Minimally invasive MV surgery in patients above 70 years may be associated with lower 30-day mortality and incidence of postoperative complications compared with sternotomy.

## Graphical Abstract

**Figure:**
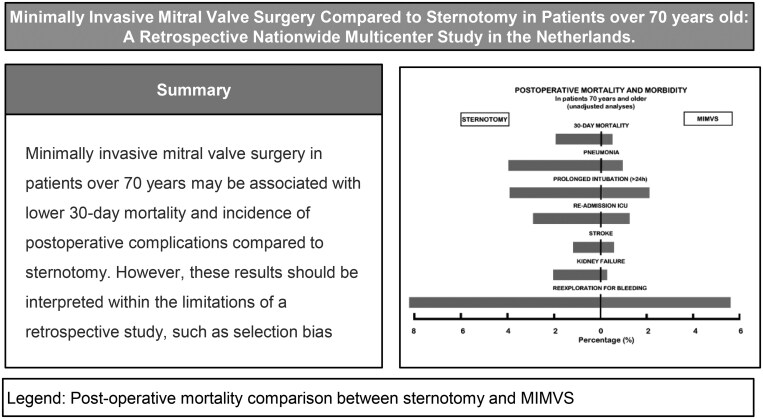

## INTRODUCTION

Older patients are more prone to postoperative morbidity and mortality after cardiac surgery.^1^ Although this excess risk is partially a result of increased comorbidities and other operative factors, age remains an independent risk factor for operative risk for surgical mitral valve (MV) procedures.^2^ Nowadays, a minimally invasive MV surgical approach (MIMVS) is increasingly adopted worldwide.^3^ Although there are no prospective studies comparing MIMVS with the traditional sternotomy approach in the elderly, the recent randomized UK mini-mitral trial showed equal repair rates and equal safety in younger patients (study mean age 67 years).^4^ Furthermore, several observational studies have demonstrated the potential benefit of MIMVS, without a compromise in the efficacy, and without increased morbidity or mortality.^5^^,^^6^ The potential benefits of MIMVS, such as reduced rate of prolonged respiratory support, reduced postoperative pain, and lower risk of wound infections, may outweigh the potential drawbacks of longer operation and aortic cross-clamp times, especially in elderly patient population.

Defining the outcomes of MV surgery in the elderly is particularly important as new (less-invasive transcatheter) therapies claim short-term superiority over traditional surgical approaches. Such advantages may be particularly applicable to older patients. However, actual benchmark outcomes for MV surgery in general, and MIMVS in particular, are scarce for elderly patients. Therefore, the aim of the current study was to compare the short-term and mid-term outcomes of patients above 70 years undergoing primary MV surgery.

## METHODS

### Source of study data

The Netherlands Heart Registration (NHR) is a prospective database that contains information on all patients undergoing cardiac surgery in the Netherlands. This database encompasses a broad range of data related to cardiac surgical procedures, such as demographic information, types of interventions, parameters related to perioperative morbidity and mortality, survival, and all necessary risk factors required to calculate risk scores. All data are anonymized, ensuring the privacy of patients, surgeons, and centres. Data collection and registration is performed by the participating centres from the electronic health records in a secured online environment, in compliance with data definitions which are described in a detailed data dictionary, available via www.nhr.nl. Detailed information on the process of data acquisition, completeness, data quality, and analysis of the NHR has been published previously.^7^^,^^8^ To obtain reliable data, the NHR has an advanced, certified data quality control system in place to ensure completeness and quality of data.^9^ Mortality data were obtained by checking the regional municipal administration registration (Basisregistratie Personen [BRP]).

### Centre characteristics

The dataset is anonymized for the researchers and the data are therefore not relatable to institutions or individual patients. In total, 16 centres provided data to the NHR. Of these 16 centres, 11 exclusively performed MV surgery through sternotomy, 3 centres almost exclusively performed MIMVS, and 2 centres performed MIMVS and median sternotomy (MST) to an equal amount. Due to the anonymized character of the data, separate centre-specific analysis could not be performed.

### Inclusion

The current study included all patients 70 years and older at the time of surgery, between January 2013 and December 2021, undergoing primary elective MV surgery through either sternotomy or MIMVS. Procedures that were considered as being part of MV surgery were concomitant procedures like tricuspid valve (TV) surgery, rhythm surgery, and closure of an atrial septal defect. All *other* concomitant cardiac procedures were excluded. To enhance a homogenous cohort, patients with endocarditis were excluded. The MIMVS was defined as right anterolateral thoracotomy avoiding sternotomy. As technical details (e.g. cannulation, fully endoscopic, robotic, aortic clamping) are not recorded in the NHR database, only details regarding the access site of MIMVS were available. To establish a predominantly homogeneous cohort, subgroup analysis was conducted focusing on truly isolated MV surgery, defined as MV surgery without any other procedure (such as TV surgery).

### Outcomes

All short-term mortality and morbidity data were retrieved from the database. The definitions of postoperative complications are summarized in **Supplementary Materials 2**. Mid-term outcomes were defined as survival at 5 years and were obtained from municipal administration records.

### Statistical analysis

Normally distributed data were presented as mean ± standard deviation (SD) while other not normally distributed continuous data were presented as median with interquartile range (IQR). Categorical data were expressed as frequencies and percentages, and the χ^2^ test was used to compare them. Fisher exact test was employed when the minimum expected cell size assumption did not apply. Kaplan-Meier survival curves were used to demonstrate 5-year survival, and the log-rank test was used to assess differences between survival curves.

The association between surgical approach (independent variable) and 30-day mortality (dependent variable) was assessed in a univariable binary logistic regression analysis. Proportionality was evaluated using the Hosmer-Lemeshow goodness-of-fit test, for which a *P* value of <.05 indicated a poor model fit. Potentially statistically important covariates (*P* < .20 based on Grant et al^10^) in the univariable analysis, and surgical approach (forced in the model, independent of the *P* value), were included in a multivariable logistic regression. To adhere to the limit of one variable per 10 events, a propensity score was computed for preoperative comorbidity consisting of age, male gender, body mass index, diabetes, chronic obstructive pulmonary disease, peripheral arterial disease, serum creatinine, reduced left ventricular ejection fraction (<50%), and pulmonary hypertension.

The influence of the surgical approach on 5-year survival was assessed using the Cox proportional hazards model. Potentially statistically important covariates (*P* < .20 based on Grant et al^10^) in the univariable analysis, and surgical approach (forced in the model, independent of the *P* value), were included in a multivariable Cox regression analysis.

Proportionality of both models (30-day mortality and 5-year survival) was evaluated using a goodness-of-fit test, in which a *P* value of <.05 demonstrated violation of the proportional hazard’s assumption (Schoenfeld residuals test). Statistical analyses of the data were performed using SPSS software (V26, IBM, Armonk, New York, United States).

### Ethical statement

The study was approved by the institutional review board MEC-U (W19.270), and written patient informed consent was waived due to the retrospective nature of this study.

## RESULTS

### Baseline characteristics

We included a total of 1429 patients A total of 805 patients underwent surgery through MST and 624 patients through MIMVS. Median age was 75.0 years (72.0-78.0) for MST and 75.0 years (72.0-79.0) for MIMVS (*P* = .12). There was no significant difference in the distribution of sex between the groups (female: MST 51.6% vs MIMVS 50.4%; *P* = .68). More details are depicted in **Table 1**. Baseline characteristics of the isolated MV subgroup are presented in **Supplementary Materials 3**.

**Table 1.** Baseline Characteristics of Patients 70 Years and Older Undergoing Mitral Valve Surgery

	Sternotomy	Minimally invasive	*P* value
	*n* = 797	*n* = 621	
Age, years median [IQR]	75.0 [72.0-78.0]	75.0 [72.0-79.0]	0.12
Female, *n* (%)	411 (51.6)	313 (50.4)	0.19
Body mass index, kg/m^2^, median [IQR]	25.0 [22.8-27.6]	24.7 [22.3-27.1]	0.11
Diabetes, *n* (%)	64 (8.0)	49 (7.9)	0.04
Chronic obstructive lung disease, *n* (%)	80 (10.0)	64 (10.3)	0.87
Peripheral vascular disease, *n* (%)	31 (3.9)	20 (3.2)	0.51
Recent myocardial infarction, *n* (%)	6 (0.8)	2 (0.3)	0.83
Serum creatine (µmol/L), median [IQR]	87.0 [74.0-103.0]	86.0 [73.9-101.0]	0.23
Left ventricular ejection fraction, median [IQR]	55.0 [50.0-55.0]	55.0 [55.0-55.0]	0.16
Pulmonary artery pressure, median [IQR]	25.0 [25.0-40.0]	25.0 [25.0-33.0]	0.88
EuroSCORE I, median [IQR]	6.3 [4.7-8.5]	6.0 [4.6-8.5]	0.41
**Surgical procedure**			
Mitral valve repair, *n* (%)	619 (77.7)	402 (64.7)	<0.01
Mitral valve replacement, *n* (%)	178 (22.3)	219 (35.3)	<0.01
**Concomitant procedures**			
Atrial septal defect closure, *n* (%)	20 (2.5)	27 (4.3)	0.06
Rhythm surgery, *n* (%)	212 (26.6)	148 (23.8)	0.24
Tricuspid valve surgery, *n* (%)	350 (43.9)	113 (18.2)	<0.01

Abbreviation: IQR, interquartile range.

### Surgical procedures

Mitral valve repair was more frequently performed in patients operated through MST (77.7% vs 64.7%; *P* < .01). Subgroup analysis for isolated MV surgery also showed a higher rate of valve repair in patients operated through sternotomy (MST 72.7% vs MIMVS 61.6%; *P* < .02). Tricuspid valve surgery was more frequently performed in MST compared with MIMVS (43.9% vs 18.2%; *P* < .01).

### Mortality

**Figure 1.**
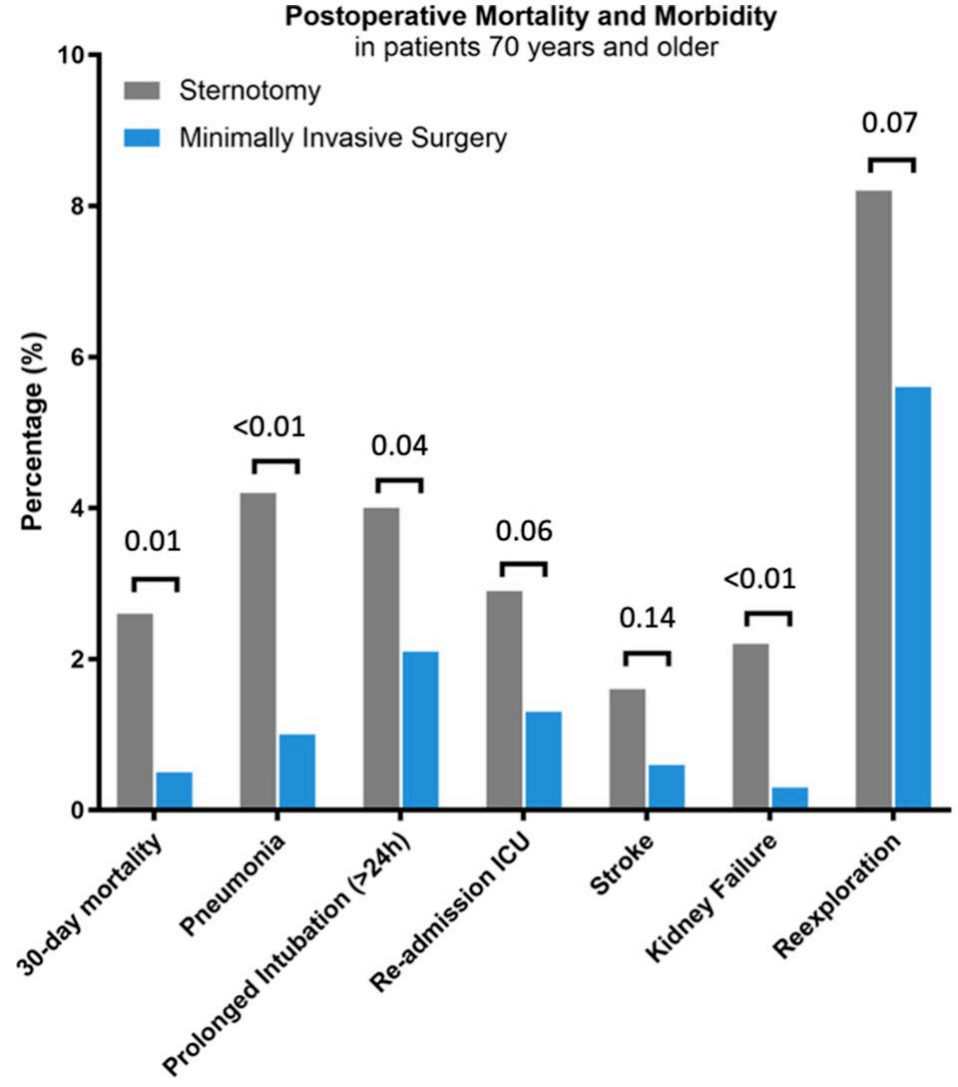
Mortality and Morbidity of Patients 70 Years and Older Undergoing Mitral Valve Surgery

**Figure 2.**
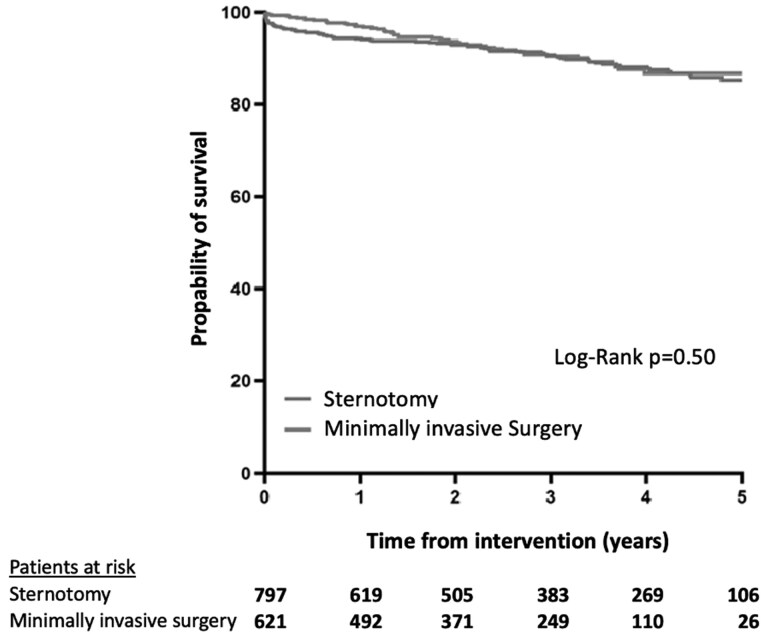
Kaplan–Meier Survival Analysis of Mitral Valve Surgery in Patients 70 Years and Older

In the overall cohort, 30-day mortality was 1.7%. A significant lower 30-day mortality was found for MIMVS compared with MST (0.6% vs 2.6%; *P* = .01) in unadjusted analysis (**Figure 1**). This difference in 30-day mortality persisted when specified to isolated MV surgery (MIMVS 0.5% vs MST 3.0%; *P* = .01) **(Supplementary Materials 4)**. Five-year survival was 89.9 ± 4.7% for MST and 91.8 ± 5.1% for MIMVS (Log-Rank *P* = .50) (**Figure 2**). In isolated MV surgery, 5-year survival was 89.1 ± 4.6% for MST and 91.6 ± 4.7% for MIMVS, Log-Rank *P* = .51 (**Supplementary Materials 5**).

Mortality was further analyzed for repair and replacement subgroups. For repair, 30-day mortality was 0.5% for MIMVS and 1.5% for MST (*P* = .22). Five-year survival was 91.1 ± 2.0% for MIMVS and 88.5 ± 1.7% for MST (Log-Rank *P* = .58).

For replacement, 30-day mortality was 0.9% for MIMVS and 6.2% for MST (*P* < .03). Five-year survival was 80.5 ± 3.6% for MIMVS and 74.5 ± 4.5% for MST (Log-Rank *P* = .155).

### Morbidity

Median length of hospital stay was 7 days in both groups. In general, lower incidence of postoperative complications were found after MIMVS. All details are depicted in **Table 2** and **Figure 1**. Postoperative complications of isolated MV surgery are summarized in **Supplementary Materials 4.** 

**Table 2.** Postoperative Mortality and Morbidity of Patients 70 Years and Older Undergoing Mitral Valve Surgery

	Sternotomy	Minimally invasive	*P* value
Mortality	*n* = 797	*n* = 621	
30-day mortality, *n* (%)	20 (2.5)	4 (0.6)	0.01
**Complications**			
Pneumonia, *n* (%)	33 (4.1)	6 (1.0)	<0.01
Urinary tract infection, *n* (%)	17 (2.1)	9 (1.4)	0.34
Reintubation due to respiratory insufficiency, *n* (%)	5 (0.6)	8 (1.3)	0.20
Prolonged intubation (>24 h), *n* (%)	32 (4.0)	13 (2.1)	0.04
Re-admission to intensive care unit, *n* (%)	22 (2.7)	8 (1.3)	0.06
Stroke, *n* (%)	13 (1.6)	4 (0.6)	0.14
Stroke with neurological deficit, *n* (%)	8 (1.0)	3 (0.5)	0.37
Stroke without neurological deficit, *n* (%)	5 (0.6)	1 (0.2)	0.24
Kidney failure, *n* (%)	16 (2.0)	2 (0.3)	<0.01
Gastrointestinal complications, *n* (%)	7 (0.9)	3 (0.5)	0.66
Vascular complications, *n* (%)	1 (0.1)	2 (0.3)	0.59
New-onset arrhythmia, *n* (%)	286 (35.9)	181 (29.1)	0.01
Re-exploration (<30 days), *n* (%)	65 (8.2)	35 (5.6)	0.07
Deep sternal wound infection, *n* (%)	3 (0.4)	0 (0.0)	0.26
Hospital stay in days, median [IQR]	7 [5-10]	7 [5-10]	0.28

Abbreviation: IQR, interquartile range.

### Risk factors for 30-day mortality

Univariable analysis of risk factors for 30-day mortality was performed and summarized in **Supplementary Materials 6.** In multivariable logistic regression analysis, MIMVS (odds ratio [OR] = 0.21; 95% confidence interval [CI] = 0.07-0.63) remained a protective factor for 30-day mortality (**Table 3**). The model has a Hosmer-Lemeshow goodness-of-fit *P* = .29. Mitral valve replacement and the propensity score of comorbidities were identified as univariable risk factors for 30-day mortality (**Table 3**).

**Table 3.** Propensity Score Logistic Regression Analysis for 30-Day Mortality in Patients 70 Years and Older Undergoing Mitral Valve Surgery

	Univariable		Multivariable	
	OR (95% CI)	*P* value	OR (95% CI)	*P* value
Minimally invasive approach	0.25 (0.09-0.74)	0.01	0.21 (0.07-0.63)	0.01
Propensity score of comorbidities^a^	0.02 (0.00-29.50)	0.28	0.04 (0.00-49.424)	0.37
Mitral valve replacement	3.11 (1.38-7.01)	<0.01	3.91 (1.72-8.89)	0.01

Abbreviations: CI, confidence interval; OR, odds ratio.

a Consists of age, male gender, body mass index, diabetes, chronic obstructive lung disease, peripheral vascular disease, serum creatinine, reduced left ventricular ejection fraction (<50%) and pulmonary artery pressure. List of individual univariable analysis is presented in **Supplementary Materials 6**.

### Mid-term outcomes

Univariable Cox regression analysis showed no statistically significant effect of surgical approach on 5-year survival. Multivariable analysis showed no statistically significant influence of surgical approach on mid-term mortality either (hazard ratio = 0.82 [0.57-1.18]) (**Table 4**). Of note, Schoenfeld residuals confirmed the validity of the proportional hazard assumption.

**Table 4.** Univariable and Multivariable Cox Regression Analysis for the Effect of Surgical Approach on Mid-Term Mortality

	Univariable			Multivariable		
	HR	95% CI	*P* value	HR	95% CI	*P* value
Minimally invasive approach	0.89	(0.63-1.27)	0.52	0.82	(0.57-1.18)	0.29
Age (years)	1.05	(1.01-1.09)	0.02	1.04	(1.00-1.09)	0.07
Sex (male)	0.85	(0.60-1.20)	0.36			
Body mass index (kg/m^2^)	1.03	(0.99-1.08)	0.12	1.02	(0.98-1.07)	0.27
Diabetes	1.88	(1.14-3.09)	<0.01	1.46	(0.86-2.48)	0.16
Chronic obstructive lung disease	2.19	(1.40-3.41)	<0.01	1.62	(1.02-2.59)	0.04
Peripheral arterial disease	2.05	(1.04-4.03)	0.04	1.47	(0.72-2.99)	0.28
Recent myocardial infarction	1.39	(0.19-9.93)	0.74			
Serum creatine (µmol/L)	1.01	(1.01-1.01)	<0.01	1.01	(1.00-1.01)	<0.01
Left ventricular ejection fraction <50%	1.72	(1.20-2.47)	<0.01	1.49	(1.02-2.16)	0.04
Pulmonary hypertension (>30 mmHg)	1.25	(0.86-1.83)	0.24			
Concomitant tricuspid valve surgery	1.15	(0.80-1.65)	0.44			
Concomitant atrial septal closure	0.92	(0.34-2.50)	0.88			
Concomitant rhythm surgery	0.84	(0.55-1.28)	0.42			
Mitral valve replacement	2.14	(1.52-3.02)	<0.01	1.91	(1.34-2.74)	<0.01

Abbreviations: CI, confidence interval; HR, hazard ratio; OR, odds ratio.

## DISCUSSION

The prevalence of MV disease is expected to increase with the ageing population, which in turn will result in an increased case load for surgeons.^11^ As MV surgery in elderly patients can be challenging due to the presence of comorbidities and frailty, MIMVS has emerged as an alternative approach with less trauma to the patient. Although our study comprised a nonrandomized comparison of approaches for MV surgery, we did find important indications for a clinically relevant benefit of MIMVS in elderly patients. However, these results should be interpreted within the limitations of a retrospective study, such as selection bias.

In earlier studies, with all age categories, no differences in mortality and morbidity for patients undergoing MV surgery through sternotomy or MIMVS were observed, in the Netherlands.^12^ However, in the current study we observed a lower 30-day mortality and reduced incidence in postoperative complications for patients 70 years and older operated through MIMVS, which seems clinically meaningful. Particularly, elderly and frail patients may lack the compensatory mechanisms that are present in younger age groups, which potentially resulted in the observed decreased short-term mortality for MIMVS. Despite a large patient population, only 24 events (30-day mortality) were observed, which limits our ability to perform robust statistical analyses. Nevertheless, we observed generally favourable outcomes in patients who underwent MIMVS, a finding that warrants attention.

The overall repair rate of our study is comparable with what is reported in literature for elderly patients.^13^ Indeed, MV repair is associated with lower 30-day mortality and long-term outcomes in several large registries,^13^ which is confirmed by this study. In our subanalysis of MIMVS versus MST, we found that 30-day mortality were similar in the repair group, but significantly different in the replacement group (0.9% for MIMVS and 6.2% for MST, *P* < .03). This could have negatively influenced the MST group as a whole. Although selection bias may have influenced our findings, we attempted to correct for this through multivariable analysis, which indicated that both MST and MVR were independent predictors of 30-day mortality. However, we acknowledge that selection bias could not be entirely eliminated. Nonetheless, despite the higher replacement rate in MIMVS, the 30-day mortality was lower, and 5-year survival was the same. Consequently, we definitely do not argue **for** MV replacement in elderly, when repair is feasible, as repair is known to be superior—even in elderly patients.^14^ On the contrary, we cannot analyze the association between MIMVS and a reduced repair rate, as the NHR did not specific MV disease aetiology (i.e. degenerative or functional). Importantly, the recent randomized UK Mini Mitral trial did not find a difference in repair rates between MIMVS and MST in patients operated on by experienced surgeons for degenerative MV disease.^4^ Still, this study also emphasizes the importance of operator experience, a known factor that contributes to repair rate and outcome in MV surgery.^15^

Several single-centre studies have shown a reduced blood loss in MIMVS with reduced need for postoperative transfusion, mainly due to smaller incision and less trauma.^16^ Consequently, transfusion of blood or blood products during cardiac surgery is associated with increased short-term and long-term mortality.^17^ Furthermore, a sternal-sparing approach may result in better preservation of the chest wall which can lead to improved respiratory function and a reduced rate of postoperative pneumonia.^18^^,^^19^ The current study found an increased incidence of all respiratory complications (i.e. pneumonia, prolonged intubation, and reintubation) in the unadjusted analyses in the MST group.

It is important to discuss the significant differences in short-term outcomes, while a long-term treatment effect of MIMVS seems to be absent. One may argue that the difference at 5 years is of most importance, and therefore both approaches may be valued equally. However, the clinically important difference in area under the curve in the first years, underlined by the difference in 30-day mortality, demonstrates the presence of more years lived in the MIMVS group, as compared with the sternotomy group. Furthermore, the relatively low numbers at risk in the final years of the Kaplan-Meier curves may contribute to a reduced statistical power of the analysis.

Finally, the current multicentre experience of unselected patients undergoing MV surgery from a nationwide registry sets a benchmark for the surgical treatment of MV disease in elderly patients. Indeed, elderly patients are progressively considered for MV interventions. Given the perceived increased periprocedural risk associated with surgical interventions, transcatheter interventions (such as percutaneous edge-to-edge repair) are on the rise, albeit with questionable long-term freedom from (severe) mitral regurgitation. Based on the current findings, and particularly in the group of elderly patients eligible for MIMVS, this technique is associated with an excellent safety profile.

### Strengths and limitations

The study has several strengths and limitations. In this broad dataset, we established an independent MIMVS treatment effect on short-term mortality, despite the presence of more risk factors in these patients (particularly MV replacement). However, patient selection is indeed an important aspect in the current retrospective study. Although the patients are at baseline comparable with no difference in risk score, frailty is particularly relevant. Depending on the experience of the surgeon, frail patients may be directed towards a classical open approach. As the extensiveness of concomitant rhythm surgery was not standardly recorded in our database, we were not able to specify which patients underwent Cox Maze, which have improved mid-term freedom from recurrent atrial fibrillation and possibly improved mid-term survival. Furthermore, concomitant surgery was more often performed through MST, suggesting that the MST group consisted of more complex patients, especially in patients with concomitant TV surgery, who are possibly at higher risk of early postoperative mortality.^20^ However, we expect that this effect is only minimal since the STS reported equal mortality in these patients.^21^ Nonetheless, we corrected for this by performing a subanalysis with isolated MV surgery and the advantages of MIMVS remained significant. Despite every attempt to adjust for all these measurable factors (repair rate, concomitant surgery, comorbidities), the risk of selection bias will always remain in retrospective studies like these and there may always be presence of unmeasurable confounders. These factors may have contributed to treatment allocation bias, in which “healthier” patients are allocated to the minimally invasive approach.^21^ Still, these limitations are partly mitigated for as the majority of centres exclusively performed MV surgery through sternotomy, implying that there is no potential of treatment allocation bias in these institutions. Unfortunately, the current study is limited by the anonymized nature of the data, which prohibits the performance of centre-specific sensitivity analyses. The NHR is an intervention-based registration, rather than a diagnosis-based registry, and no data regarding disease aetiology and severity of the MV and TV disease was available. Therefore, we were not able to perform an in-depth analysis of repair rates and concomitant TV surgery. The same applies to perioperative conversion from MIMVS to MST, given that these patients are expected to have a more complicated course. Length of stay is expected to be lower in the mini-group,^4^ and we found no difference in our study. Discharge protocols may differ between hospitals (e.g. fast track), which could have played a role. Finally, MV replacement has been associated with impaired outcome, as compared with MV repair. Still, we found a beneficial effect of MIMVS in both unadjusted and adjusted analyses, despite the higher incidence of replacement in MIMVS patients, emphasizing the robustness of our results.

## CONCLUSION

Minimally invasive MV surgery in patients above 70 years is associated with lower 30-day mortality and morbidity compared with MST. Although we observed no difference in the 5-year survival rate between both groups, the beneficial short-term treatment effect may be of particular interest to elderly patients. Still, our findings should be interpreted within the inherent limitations of the study design, such as selection bias. Nevertheless, we provide a benchmark for the surgical treatment of MV disease in the elderly.

## Supplementary Material

ivag026_Supplementary_Data

## Data Availability

Data will be shared upon reasonable request to the corresponding author.
