# Supplementary material for: Minimally Invasive Mitral Valve Surgery Compared to Sternotomy in Patients Over 70 Years Old: A Retrospective Nationwide Multicentre Study in The Netherlands
Source: Interdiscip Cardiovasc Thorac Surg. 2026 Jan 28;41(2):ivag026. doi: 10.1093/icvts/ivag026 (PMC12881947; doi:10.1093/icvts/ivag026)
Supplement: ivag026_Supplementary_Data [file ivag026_supplementary_data.zip › supplement 3.0.docx]

APPENDIX

Supplementary materials 1

Cardiothoracic Surgery Registration Committee of the Netherlands Heart Registration

Dr. S. Bramer Amphia

Dr. R.A.F. de Lind van Wijngaarden Amsterdam UMC, locatie AMC

Drs. B.M.J.A. Koene Catharina Ziekenhuis

Dr. J.A. Bekkers Erasmus MC

Dr. G.J.F. Hoohenkerk HagaZiekenhuis

Dr. A.L.P. Markou Isala

Drs. A. de Weger Leids Universitair Medisch Centrum

Dr. P. Segers Maastricht UMC+

Drs. D. Stecher Medisch Centrum Leeuwarden

Dr. R.G.H. Speekenbrink Medisch Spectrum Twente

Dr. W. Stooker OLVG

Drs. W.W.L. Li Radboudumc

Drs. E.J. Daeter St. Antonius Ziekenhuis

Dr. N.P. van der Kaaij UMC Utrecht

Dr. Y.L. Douglas Universitair Medisch Centrum Groningen

Supplementary Material 2 – Definitions of postoperative complications

*Pneumonia*

Lung infection/pneumonia with positive sputum culture or treatment

*Urinary tract infection*

Infection with positive urine culture.

*Reintubation due to respiratory insufficiency*

Respiratory failure requiring reintubation.

*Prolonged intubation (>24 hours)*

Ventilation for more than 24 hours.

*Readmission to ICU*

Readmission to the Intensive Care Unit (ICU) or Post Anesthesia Care Unit (PACU) after initial discharge from the IC/PACU. This does not include a stay in the Medium Care (MC).

*Stroke*

The combined endpoint of stroke without neurological deficit and stroke with neurological deficit

*Stroke without neurological deficit*

A neurologist has determined that a central neurological deficit (CVA) during

the postoperative period has occurred, but with no residual injury at discharge.

This also concerns a Transient Ischemic Attack (TIA)

*Stroke with neurological deficit*

A neurologist has determined that a postoperative stroke during the

hospitalization of the current intervention has occurred (excluding TIA).

CVA = permanent neurological dysfunction diagnosed by a neurologist as

due to focal ischemia of the brain, spinal cord, or retina, caused by

an acute infarction of the neurological tissue due to thrombosis, embolism,

systemic hypoperfusion or bleeding.

*Kidney failure*

Renal failure occurs if one or more of the following STS criteria are met during the postoperative period

• Renal replacement therapy (dialysis, CVVH) which was not initiated preoperatively

• Highest postoperative creatinine value > 177 µmol/L and doubling of the preoperative value (as preoperative value: the value of the creatinine on which the EuroSCORE is calculated).

*Gastro-intestinal complications*

Bleeding: gastrointestinal bleeding requiring therapy such as transfusion,

scope or surgery.

Other: intestinal ischemia, acalculous cholecystitis.

*Vascular complications*

The occurrence of any vascular complications during hospitalization,

diagnosis according to the VARC-2 definitions, from the start of the current intervention

(including peroperative vascular complications and excluding stroke).

*New-onset arrhythmia*

All forms of de novo rhythm problems requiring treatment (such as

resuscitation in connection with asystole, new onset atrial fibrillation / flutter for which

specific intervention (defibrillation, medication) is necessary). Below is not

considered: a spontaneously transient period of atrial fibrillation, without any

consequence for the patient.

Mediastinitis

Deep sternal wound infection (mediastinitis) within 30 days. Includes muscle, sternum,

mediastinum and is positive if one or more of the following criteria is present:

• Surgical drainage / sternum refixation in deep sternum wound infection

• Positive wound cultures.

• AB therapy due to the sternum wound.

This includes a deep sternal wound infection that occurred after the patient

was discharged from that hospital.

*Reexploration (within 30 days)*

Rethoracotomy within 30 days due to a complication of the current intervention. This

also includes rethoracotomies performed after the patient has been discharged.

This concerns the first rethoracotomy after the initial closing of the thorax. This

applies to all causes, with the exception of opening the sternum in due to

mediastinitis or refixation of the sternum.

Supplementary Material 3 - Baseline characteristics of isolated mitral valve surgery

|  |  |  |  |
| --- | --- | --- | --- |
|  | Sternotomy | Minimally invasive surgery | P-value |
|  | n= 333 | n= 380 |  |
| Age (years), median [IQR] | 75.0 [72.0 - 78.0] | 75.0 [72.0 - 79.0] | 0.34 |
| Female, n (%) | 169 (50.8) | 185 (48.7) | 0.58 |
| BMI (kg/m^2^), n (%) | 25.3 [23 - 28.] | 25 [23 - 27.0] | 0.03 |
| Diabetes, n (%) | 29 (8.7) | 30 (7.9) | 0.69 |
| COPD, n (%) | 28 (8.4) | 42 (11.1) | 0.24 |
| PAD, n (%) | 15 (4.5) | 13 (3.4) | 0.46 |
| Recent myocardial infarction, n (%) | 0 (0) | 1 (0.3) | 0.99 |
| Serum creatine (µMol/L), median [IQR] | 85 [72 - 101] | 85 [72 - 100] | 0.82 |
|  |  |  |  |
| LVEF, median [IQR] | 55.0 [55.0 - 60.0] | 55.0 [55.0 - 55.0] | 0.02 |
| PAP, median [IQR] | 25.0 [25.0 - 25.0] | 25.0 [25.0 - 25.0] | 0.42 |
|  |  |  |  |
| EuroSCORE I, median [IQR] | 6.2 [4.4 - 8.3] | 6.0 [4.6 - 8.7] | 0.45 |
|  |  |  |  |
| **Surgical procedure** |  |  |  |
| Mitral valve repair, n (%) | 242 (72.7) | 234 (61.6) | **0.02** |
| Mitral valve replacement, n (%) | 91 (27.3) | 146 (38.4) | **0.02** |
|  |  |  |  |
|  |  |  |  |
| **Concommitant procedures** |  |  |  |
| Atrial Septal Closure, n (%) | - | - | - |
| Rhythm surgery, n (%) | - | - | - |
| Tricuspid valve surgery, n (%) | - | - | - |

BMI: Body Mass Index, COPD: Chronic Obstructive Lung Disease, PAD: Peripheral Vascular Disease, LVEF: Left ventricular Ejection Fraction, PAP: Pulmonary Artery Pressure

Supplementary Material 4 – Postoperative complications of isolated mitral valve surgery

|  | Sternotomy | | | |  | | Minimally invasive | | | | P-value | |  |
| --- | --- | --- | --- | --- | --- | --- | --- | --- | --- | --- | --- | --- | --- |
| **Mortality** | | n=333 | |  | |  | | n=380 | |  | |  | |
| 30-day mortality, n (%) | 10 | | (3,0) | |  | | 2 | | (0,5) | | 0,010 | |  |
|  |  | |  | |  | |  | |  | |  | |  |
| **Complications** |  | |  | |  | |  | |  | |  | |  |
| Pneumonia, n (%) | 8 | | (2,4) | |  | | 4 | | (1,1) | | 0,162 | |  |
| Urinary tract infection, n (%) | 8 | | (2,4) | |  | | 7 | | (1,8) | | 0,603 | |  |
| Reintubation due to respiratory insufﬁcency, n (%) | 1 | | (0,3) | |  | | 3 | | (0,8) | | 0,383 | |  |
| Prolonged intubation (>24 h), n (%) | 7 | | (2,1) | |  | | 6 | | (1,6) | | 0,602 | |  |
| Re-admission to ICU, n (%) | 6 | | (1,8) | |  | | 3 | | (0,8) | | 0,227 | |  |
| Stroke, n (%) | 7 | | (2,1) | |  | | 2 | | (0,5) | | 0,060 | |  |
| Stroke with neurological defecit, n (%) | 4 | | (1,2) | |  | | 2 | | (0,5) | | 0,325 | |  |
| Stroke without neurologcial defecit, n (%) | 3 | | (0,9) | |  | | 0 | | (0,0) | | 0,064 | |  |
| Kidney failure, n (%) | 5 | | (1,5) | |  | | 2 | | (0,5) | | 0,188 | |  |
| Vascular complications, n (%) | 0 | | (0,0) | |  | | 1 | | (0,3) | | 0,349 | |  |
| New-onset arrhythmia, n (%) | 137 | | (41,1) | |  | | 97 | | (25,5) | | 0,000 | |  |
| Reexploration (within 30 days), n (%) | 19 | | (5,7) | |  | | 19 | | (5,0) | | 0,676 | |  |
| Deep sternal wound infection, n (%) | 0 | | (0,0) | |  | | 0 | | (0,0) | | 1,000 | |  |
|  |  | |  | |  | |  | |  | |  | |  |
| Hospital stay in days, median [IQR] | 6.0 | | [4 - 8] | |  | | 6.0 | | [4 - 8] | | 0,609 | |  |

ICU: Intensive Care Unit

Supplementary materials 5 Kaplan Meier Survival analysis of **isolated** mitral valve surgery in patients 70 years and older.


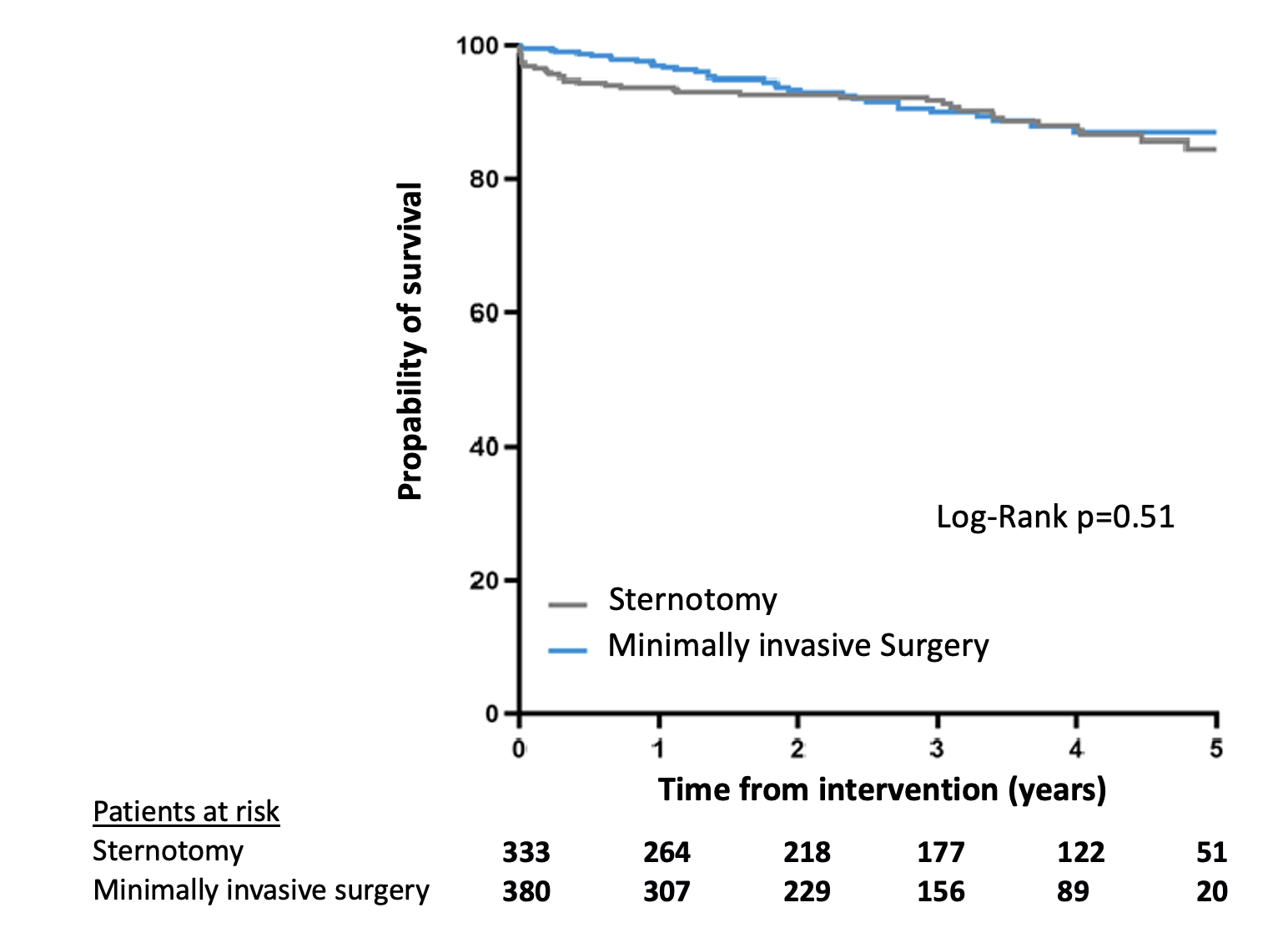


Supplementary materials 6 Univariate analysis for 30-day morality in patients 70 years and older undergoing mitral valve surgery.

|  | Univariate |  |  |
| --- | --- | --- | --- |
|  | **OR** | **95% CI** | **p value** |
| **Minimally invasive approach** | 0,25 | (0,09-0,74) | **0,01** |
|  |  |  |  |
| **Age (years)** | 1,07 | (0,97-1,18) | 0,17 |
| **Sex (male)** | 1,94 | (0,83-4,56) | 0,12 |
| **BMI (kg/m^2^)** | 1,14 | (1,05-1,23) | **<0.01** |
|  |  |  |  |
| **Diabetes** | 1,71 | (0,5-5,83) | 0,38 |
| **COPD** | 3,05 | (1,19-7,8) | **0,01** |
| **PAD** | 1,00 | (1,00-1,00) | 0,99 |
| **Recent myocardial infarction** | 8,58 | (1,01-72,62) | **0,02** |
| **Serum creatine (µMol/L)** | 1,01 | (1,00-1,02) | **<0.01** |
|  |  |  |  |
| **Left ventricular Ejection Fraction <50%** | 1,50 | (0,62-3,65) | 0,37 |
| **Pulmonary hypertension( PAP >30mmHg)** | 2,69 | (1,20-6,05) | **0,01** |
|  |  |  |  |
| **Concomitant Tricuspid valve surgery** | 0,86 | (0,35-2,08) | 0,73 |
| **Concomitant Atrial Septal Closure** | 1,00 | (1,00-1,00) | 0,99 |
| **Concomitant Rhythm surgery** | 0,77 | (0,28-2,07) | 0,60 |
|  |  |  |  |
| **Mitral valve replacement** | 3,12 | (1,38-7,01) | **<0.01** |

BMI: Body Mass Index, COPD: Chronic Obstructive Lung Disease, PAP: Pulmonary Artery Pressure
